# An Ultra Scale-Down Analysis of the Recovery by Dead-End Centrifugation of Human Cells for Therapy

**DOI:** 10.1002/bit.25519

**Published:** 2015-03-12

**Authors:** M Delahaye, K Lawrence, S J Ward, M Hoare

**Affiliations:** 1 Department of Biochemical Engineering, Advanced Centre for Biochemical Engineering, University College LondonTorrington Place, London, WC1E 7JE, UK; 2 Onyvax Ltd., St. Georges Hospital Medical SchoolLondon, SW17 0RE, UK

**Keywords:** ultra scale-down, human cells, cell therapy, centrifugation, cell quality

## Abstract

An ultra scale-down method is described to determine the response of cells to recovery by dead-end (batch) centrifugation under commercially defined manufacturing conditions. The key variables studied are the cell suspension hold time prior to centrifugation, the relative centrifugal force (RCF), time of centrifugation, cell pellet resuspension velocities, and number of resuspension passes. The cell critical quality attributes studied are the cell membrane integrity and the presence of selected surface markers. Greater hold times and higher RCF values for longer spin times all led to the increased loss of cell membrane integrity. However, this loss was found to occur during intense cell resuspension rather than the preceding centrifugation stage. Controlled resuspension at low stress conditions below a possible critical stress point led to essentially complete cell recovery even at conditions of extreme centrifugation (e.g., RCF of 10000 g for 30 mins) and long (∼2 h) holding times before centrifugation. The susceptibility to cell loss during resuspension under conditions of high stress depended on cell type and the age of cells before centrifugation and the level of matrix crosslinking within the cell pellet as determined by the presence of detachment enzymes or possibly the nature of the resuspension medium. Changes in cell surface markers were significant in some cases but to a lower extent than loss of cell membrane integrity. Biotechnol. Bioeng. 2015;112: 997–1011. © 2014 Wiley Periodicals, Inc.

## Introduction

The capacity to bring new whole cell therapies and regenerative medicines to a wide range of patients will ultimately rest on the ability to process large numbers of cells either by scale up or scale out routes to manufacture (Brandenberger, 2011; Seth et al., 2006; Want et al., 2012; Zoro et al., 2008). Within a bioprocessing sequence the cell product will undergo a series of stages that will involve exposure to stress which may lead to adverse effects on cell quality, for example, membrane leakage (Barbee, 2005; Dhondalay et al., 2014; Ma et al., 2002; McCoy et al., 2009; McCoy et al., 2010), physiological and metabolic changes (Al-Rubeai et al., 1995), lysis, apoptosis or necrosis (Mollet et al., 2007; Tanzeglock et al., 2009). One requirement of cell bioprocessing is the need to recover the cells, without damage, from solution, for example, to remove growth or storage medium or to concentrate cells for administration in low volumes or for mixing with a scaffold agent in tissue preparation.

The most common method for separation, both during expansion seed trains and final product harvest stages, is to pellet the cells by dead-end (batch) centrifugation and then to resuspend the cells into a specified medium. While this method is not amenable to fully-enclosed operation (e.g., use of sterile hoods is required for transfer stages) it does provide the basis for an easily accessible process which may be used over a wide range of the relatively small scales relevant to the manufacture of cells for therapy (Pattasseril et al., 2013). It also benefits from use of low hold up volumes and the option to deliver cells at a wide range of concentrations including cell pastes. Alternative separation systems which allow fully-enclosed operation will be discussed later.

Bench-scale processes for cell preparation generally use batch dead-end centrifugation operating at a low relative centrifugal force for short times for cell recovery, for example, 500–1000*xg* for 3–6 mins (Dar et al., 2002; Pollock et al., 2006). It is expected that the stress on the cells may be reduced by the use of such conditions but a sizeable fraction of the population may be lost by their failure to pellet (Katkov & Mazur, 1999), that is, care is required to remove the supernatant from the loose sediment without resuspending the cells. A typical manufacturing process might employ a similar strategy (Lapinskas, 2010) with multiple centrifugation and resuspension steps needed to improve removal of soluble contaminants (e.g., cell metabolites, serum based proteins, and remaining growth factors).

High levels of compaction are of interest where greater extents of soluble contaminant removal are required to reduce number of wash stages and hence processing time and also where high cell densities (∼100 × 10^6^ cells/mL) are required to mix with a matrix scaffold for tissue formation (Dar et al., 2002). The use of high relative centrifugal forces will lead to the formation of compacted pellets; however the resuspension of these may expose cells to high levels of mechanical agitation, leading to a loss in cell integrity (Katkov & Mazur, 1998). For example, attempts to quantify cell recovery during centrifugation indicated 20 +/− 13% loss of cells which was not accountable as cells lost in the supernatant or as cells attached to surfaces (Zoro et al., 2009).

In this study we seek to evaluate dead-end centrifugation as a means of cell recovery and concentration and the effects upon cell quality as a result of the relative centrifugal force and time of centrifugation used. The cell lines studied are candidates for a cancer vaccine therapy (Eaton et al., 2002; Ward et al., 2008) where the processing challenges are as for cell therapy preparation in general. A selection of operating variables as might determine the performance of dead-end centrifugation is studied using an ultra scale-down approach. This is to allow the exposure of small quantities of cells to various combinations of defined operating conditions over ranges both within and outside those normally used at the full scale and in this way to gain an understanding of processing effects which may lead to cell loss, and conversely operating regions where acceptable performance might be gained.

## Materials and Methods

### Cell Preparation

Two cell line candidates for a cancer vaccine therapy, OnyCap23 and P4E6 (Onyvax Ltd, London, UK, passage number range 51–63) were cultured to 70–80% confluency (T175 flasks, Greiner Bio-One, Germany) in complete growth medium (CGM; keratinocyte serum-free medium with epidermal growth factor at a final concentration of 5 ng/mL, both Invitrogen, Paisley, UK and 2% [v/v] fetal calf serum, FCS; Thermo Fisher Scientific, Northumberland, UK); see (Acosta-Martinez et al., 2010) for details. OnyCap23 was clonally derived using the PNT2-C2 prostate cell line transformed by SV40 (Berthon et al., 1995) and P4E6 was derived from primary culture of an early prostate cancer biopsy (Maitland et al., 2001). Cell harvest was by decantation to remove spent growth medium, cell incubation in 5 mL TrypLE Select solution per flask (Invitrogen) for 6–8 min at 37°C, quenching in 5 mL CGM, centrifugation at 500*g*, 3 min, 21°C (Heraeus Multifuge X3R, Thermo Fisher Scientific, Basingstoke, UK), and cell resuspension in ∼10 mL CGM to yield a suspension of 2 × 10^6^ cells/mL at 21°C. The cells were used within 5 min for centrifugation studies. Variables in cell preparation included: resuspension to a cell concentration of 1 × 10^6^ cells/mL by dilution in CGM; replacement of CGM with HBSS free of Ca^2+^ and Mg^2+^ (Hank's balanced salt solution; Sigma–Aldrich, Ayrshire, UK) for cell resuspension; and controlled holds at 21°C for 120 min of the cells before use for centrifugation studies.

### Centrifugation Studies

**Figure 1:**
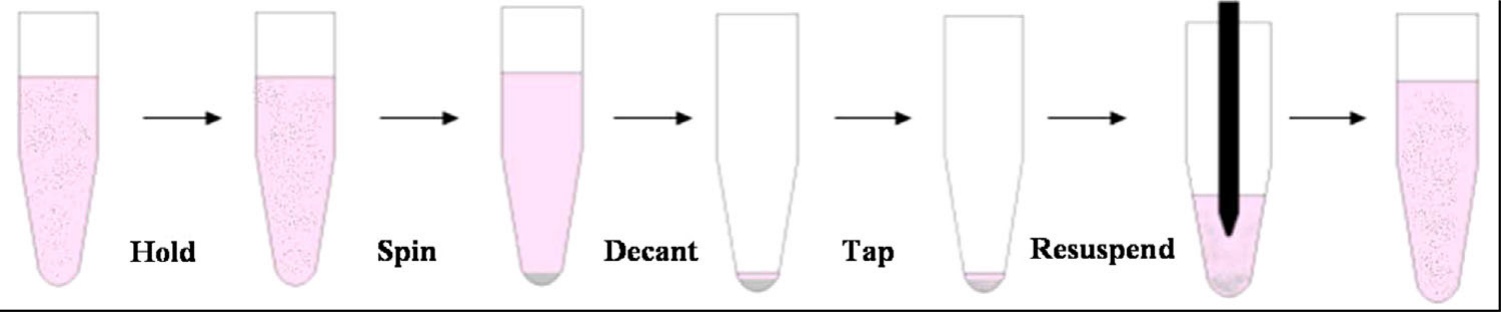
Sequence of processing stages used in centrifugation and resuspension studies (not to scale)—see text for detail. Stage 5 shows tube contents with suspending liquor withdrawn into pipette tip and about to be reinjected into suspension. For manual resuspension this occurs in a few (ca 10) high flow velocity cycles. For automated resuspension this occurs in many (ca 20–100) low velocity cycles.

The extent of cell concentration achieved as a result of dead-end centrifugation was studied using 0.5 mL aliquots of cell suspension exposed to RCF of 200 to 20000*xg* for 1–30 min at 21°C (VoluPac tubes, Sartorius, Surrey, UK in 5430 R, Eppendorf, Cambridge, UK). The effect of recovery by centrifugation on the properties of the resultant resuspended cells was studied for a fixed method of cell resuspension (see Fig. 1 for sequence of operations making up this procedure). Centrifugation of 1 mL aliquots of cell suspension was by exposure to RCF of 250 to 20000*xg* for 3–30 min at 21°C (5430R, Eppendorf). The manual resuspension method was by removal and retention of the cell supernatant, tapping (∼2 to 3 times) of the centrifuge tube until the pellet is visibly detached from the tube sides, use of the retained supernatant to resuspend the cell pellet using a 1.0 +/ − 2.5% mm id tip pipette (Gilson Scientific Ltd, Luton, UK) located ∼5 mm above the sediment surface operated in injection/suction mode timed separately at ∼2 passes per second for 10 passes (mean velocity at tip 2.5 m/s +/− 10%). Additional passes were used (<10) in the few cases where visible clumps remained. An alternative method of resuspension was by means of an electronic multichannel pipette (EDP®3, Rainin, CA) fitted with 1.0 +/− 2.5% mm id tips and located as above and programmed, based on initial observations of cell dispersion, to withdraw and redispense over a period of ∼2 min for 110 times, 500 μL of suspension at 500 μL/s (0.65 m/s). Where enzymatic treatment was used to aid resuspension, supernatant was removed and 500 μL retained for later resuspension. TrypLE Select, 500 μL, was pre-warmed to 37°C and added to cover a cell pellet and incubated at 21°C for 15 mins. For manual resuspension as above, the TrypLE Select was removed and mixed with the original supernatant, the pellet loosened by tapping and the TrypLE Select/supernatant mix used for resuspension. For electronic resuspension as above, the retained supernatant was added to the TrypLE Select to provide the resuspension medium.

The effect of the number of resuspension passes was studied using cell pellets prepared in multiwell plates (96 deepwell 2 mL round-bottomed, circular cross-section wells, Starlab Ltd., Milton Keynes, UK) filled with 0.7 mL/well of a 2 × 10^6^ cells/mL suspension and centrifuged at either 1500*xg* or 2500*xg* for 5 min (Heraeus Multifuge). Resuspension was by controlled aspiration cycles using an automated platform (Freedom Evo®75;Tecan, Männedorf, Switzerland) with pipette tips of 0.76 mm id, located 4.57 mm (i.e., 6× id) above the pellet surface and a dispensing flow rate of either 600 μL/s (1.3 m/s) or 900 μL/s (2.0 m/s).

### Analysis of Cell Suspension

Cells were counted and analysed for number, integrity and size (Vi-CELL XR™ automated analyser Beckman Coulter, High Wycombe, UK). The system utilizes the trypan blue exclusion method on a basis of image capture (50 images) and their subsequent analysis. Size analysis is achieved by measurement of the equivalent spherical diameter of all imaged cells.

Samples of processed cells were analysed for surface marker expression within 7 days of freezing. Samples were thawed (∼3 min, 37°C), centrifuged and the cells resuspended in DPBS, 0.1% w/v BSA and 0.01% w/v NaN_3_ to 5 × 10^6^ total cells/mL. Aliquots, 40 µL, were washed twice with 100 μL/well of DPBS-BSA-NaN_3_ solution, incubated (4°C, 20 min) with selected mouse anti-human antibodies, CD9, CD81, and CD147 (1:40 dilution), CD44 and MHC 1 (1:300), and CD59 (1:20) (BD BioSciences, Oxford, UK). IgG1 (clone MOPC-21) and IgG2a (clone G155–178) monoclonal isotypes were used as controls. The labeled cells were washed twice with 100 μL DPBS-BSA-NaN_3_ and incubated (4°C, 20 min) in the dark with a 1:20 dilution of goat anti-mouse antibody (BD BioSciences). The resultant cells were washed twice (100 μL DPBS-BSA-NaN_3_) and resuspended in 200 μL of FACS flow and analyzed (Epics XL MCL Flow Cytometer, Beckman Coulter calibrated using QIFIKIT beads, Dako Ltd. UK, Ely, UK coated with different, but well-defined, quantities of the respective mouse monoclonal antibodies to generate a calibration curve for the processed cells labeled to saturation with primary mouse monoclonal antibodies; a secondary fluorescent polyclonal goat anti-mouse immunoglobulin is used to track mouse monoclonal antibody binding). Mean fluorescence intensity (MFI) values were analysed (WinMDI Software, FACS Core Facility, Scripps, CA) and recorded as number of antigenic sites per cell specimen.

### Design of Experiment (DoE) studies

Selected ranges of three operating variables, hold time of the cells before centrifugation, centrifugation RCF and time, were studied using a defined DoE protocol at low, mid, and high points using the manual resuspension methods described above and analysis of the cell membrane integrity and total cell count before and after centrifugation and resuspension. All measurements of each run were carried out in triplicate and the results were analysed using the DoE software (Design Expert v7, Stat-Ease, Minnaeapolis, MN)

## Results

### Cell Compaction Studies

**Figure 2:**
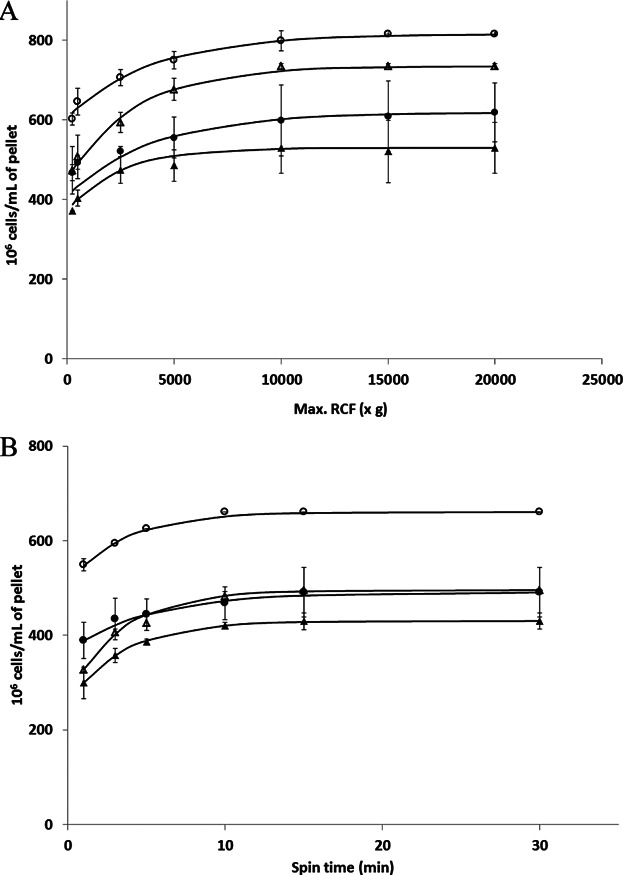
Cell concentration in a centrifuged pellet as a function of spin time and RCF for OnyCap23 (•, ▴) and for P4E6 (○, Δ). The cell concentration values are calculated from the sediment volumes derived from known numbers of cells presented for centrifugation. Trends shown are with respect to (A) changing maximum RCF for a constant spin time of 3 min (▴, Δ) or 30 min (•, ○) and (B) changing spin time for constant maximum RCF of 250*xg* (▴, Δ) or 2500*xg* (•, ○). Results are means +/− sd of separate centrifugation studies (*n* = 3); lines are best fit by eye. Initial concentration used was 2 × 10^6^ cells/mL.

Dead-end centrifugation processes for cell preparation generally use a low RCF for short times yielding loosely compacted cell suspensions. As discussed earlier, high levels of compaction are of interest to reduce washing stages and for cell preparation for tissue formation (Dar et al., 2002). To set the boundaries for the dead-end centrifugation studies in this investigation the impact of centrifugal force and spin time on the volume of sediment for a fixed number of cells was determined (Fig. 2). The cell concentrations achieved for a spin time of 3 or 30 min increased up to values of RCF ∼20000*xg* with a ∼30% increase compared to that achieved at ∼500*xg* (Fig. 2A). Similarly the concentrations achieved for a RCF of 250 or 2500*xg* increased up to spin times of 30 min with a 20% increase compared with centrifugation for 3 min (Fig. 2B). The resultant effect is up to a doubling in cell concentration being achieved for the most extreme centrifugation conditions studied as compared with those conditions commonly used at bench scale. The same trends were observed for both cell lines studied but with considerably greater concentration being obtained for P4E6 as compared with OnyCap23 cells. Further analysis of the extents of cell concentration (and hence cell sediment dewatering) achieved are presented in the Discussion section. For all centrifugation conditions studied in Figure 2, complete cell removal from the suspension was recorded by cell count analysis of the sample supernatant (data not shown).

### Ultra Scale-Down Analysis of Centrifugation Conditions

Studies to evaluate the effect of centrifugation conditions on cell quality were carried out for the full range of conditions used in Figure 2, that is, RCF of 250 to 20000*xg* for 3 to 30 min. In addition a third variable studied was the hold time of the cells after detachment from the growth surface and before centrifugation, this ranging from 5 to 120 min. The former time is as might traditionally be achieved at bench scale and the latter is more typical of full-scale processing (e.g., Onyvax private communication for a process involving the harvest of sixteen 40 layer cell factories yielding ∼20 L suspension per factory).

Three performance factors are used to characterize the effect on the cells of the combinations of operating conditions used. The retention of cell membrane integrity, V, of the resultant cell suspension relevant to the original cells is given by *V* = 100(C_out_/C_in_) where C is % of cells which are viable (in terms of the membrane integrity remaining intact with respect to trypan blue exclusion), and the subscripts “in” and “out” refer to before and after combined operations of centrifugation and resuspension. The yield of total cell numbers, Y, is given by *Y* = 100(T_out_/T_in_) where T is the total number of cells recorded, whether viable or non-viable. The key summarized performance factor is given by the % total recovery, R_EC_, of cells with intact membrane:


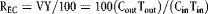
(1)

The experimental design of experiments construction along with all the results achieved are summarized in Table I with “l,” “m,” and “h” used to represent the low, midpoint, and high end of ranges used for the hold time before centrifugation, the RCF, and the spin time. Controls carried out using gravity settling for a time equal to the combined hold and spin times showed no significant loss of cell membrane integrity, yield or overall recovery of cells with an intact membrane (results not shown here). Some observations from Table I include: (i) a good level of reproducibility in the three repeat samples carried out at the medians of the ranges studied (mmm); (ii) full recovery of intact cells at the mildest centrifugation conditions studied (*RCF* = 250*xg* for 3 min spin time) especially for freshly harvested cells (lll); (iii) the lowest values of cell recovery are for extreme centrifugation conditions (*RCF* = 20000*xg* for 30 min spin time) for aged cells (hhh); (iv) greater cell losses for lower concentration feed stocks; (v) a greater susceptibility to cell loss when processing OnyCap23 as compared with P4E6; (vi) the effect of hold time before centrifugation is to increase loss. For P4E6 cells the main reason for lower levels of recovery of intact cells, R_EC_, appears to be due to loss of cell membrane integrity, V, rather than loss of yield, Y, while for OnyCap23 low levels of loss are associated with loss of cell quality while high loss levels are associated with loss of both cell membrane integrity and cell yield. This loss of yield is attributed to cell fragmentation rather than adherence to the surfaces of the centrifuge tube or pipette tip (studies using TrypLE Select suspension for 10 min to detach any remaining cells showed no increase in yield- results not shown here).

**Table I:** Operation and results of Design of Experiment studies to examine effects of relative centrifugal force (R), spin time (S), and hold time prior to centrifugation (H)

			P4E6, 2x10^6^ cells/mL, CGM								OnyCap23, 1x10^6^ cells/mL, CGM							
R	S	H	Run	C_in_	T_in_	C_out_	T_out_	V	Y	R_EC_	Run	C_in_	T_in_	C_out_	T_out_	V	Y	R_EC_
l	l	l	6	96	1.95	96	1.95	100	100	100 ± 0	2	96	1.95	95	1.95	100	100	100 ± 0
l	l	h	7	96	1.95	96	1.95	100	100	100 ± 0	4	96	1.95	96	1.95	100	100	100 ± 0
l	h	l	11	97	2.05	95	2.05	98	100	98 ± 0	8	98	2.03	98	2.03	100	100	100 ± 0
l	h	h	9	97	2.05	96	2.05	99	100	99 ± 0	7	98	2.03	97	2.03	99	100	99 ± 0
h	l	l	4	95	2.06	95	2.05	100	99	99 ± 1	3	96	1.95	93	1.93	97	99	96 ± 1
h	l	h	2	95	2.06	94	2.06	99	100	99 ± 1	10	98	1.98	95	1.97	97	99	96 ± 1
m	m	m	1	95	2.06	92	2.04	97	99	96 ± 1	1	96	1.95	91	1.93	95	99	94 ± 3
m	m	m	3	95	2.06	94	2.04	99	99	98 ± 0	9	98	1.98	96	1.96	98	99	97 ± 5
m	m	m	5	96	1.95	93	1.95	97	100	97 ± 1	6	98	2.03	94	2.01	96	99	95 ± 1
h	h	l	10	97	2.05	92	2.03	95	99	94 ± 3	5	98	2.03	89	1.98	91	97	89 ± 3
h	h	h	8	96	1.95	88	1.92	92	98	90 ± 1	11	98	1.98	53	1.69	54	85	46 ± 3

			P4E6, 2x10^6^ cells/mL, CGM								OnyCap23, 1x10^6^ cells/mL, CGM							
R	S	H	Run	C_in_	T_in_	C_out_	T_out_	V	Y	R_EC_	Run	C_in_	T_in_	C_out_	T_out_	V	Y	R_EC_
l	l	l	6	96	1.03	96	1.03	100	100	100 ± 0	9	97	1.01	97	1.01	100	100	100 ± 0
l	l	h	11	96	0.99	96	0.99	100	100	100 ± 0	5	96	0.98	94	0.95	98	97	95 ± 7
l	h	l	2	94	0.95	94	0.95	100	100	100 ± 0	8	96	0.98	96	0.98	100	100	100 ± 0
l	h	h	5	96	1.03	95	1.03	99	100	99 ± 1	10	97	1.01	92	0.95	95	94	89 ± 2
h	l	l	3	94	0.95	93	0.95	99	100	99 ± 1	6	96	0.98	93	0.97	97	99	96 ± 2
h	l	h	8	96	1.03	96	1.03	100	100	100 ± 0	3	98	1.04	92	1.04	94	100	94 ± 3
m	m	m	9	96	0.99	92	0.99	96	100	96 ± 1	4	98	1.04	90	0.82	92	79	72 ± 3
m	m	m	10	96	0.99	93	0.98	97	99	96 ± 2	7	96	0.98	86	0.80	90	82	73 ± 6
m	m	m	1	94	0.95	92	0.94	98	99	97 ± 1	11	97	1.01	84	0.83	87	82	71 ± 4
h	h	l	4	94	0.95	87	0.95	93	100	93 ± 1	1	98	1.04	85	0.91	87	88	76 ± 3
h	h	h	7	96	1.03	86	1.02	90	99	89 ± 2	2	98	1.04	67	0.67	68	64	44 ± 5

Ranges used are, for h, m, l, *R* = 20000, 10125, 250*xg* (note for OnyCap23 1 × 10^6^ cells/mL *R* = 11350, 5800, 250*xg*), *S* = 30, 16.5, 3 min, *H* = 120, 62.5, 5 min. The results are reported in terms of total cell number, T × 106/mL, cell membrane integrity, C %, retention of cell membrane integrity V %, cell yield Y%, and recovery of intact cells REC% (see equation 1). All experiments were for each centrifugation sample measured in triplicate. Only mean values for T, C, and V presented with outliers removed as defined by data external to range +/− 1 sd. Values for REC are referred as mean +/− sd (*n* = 3). See text for details of controls.

**Figure 3:**
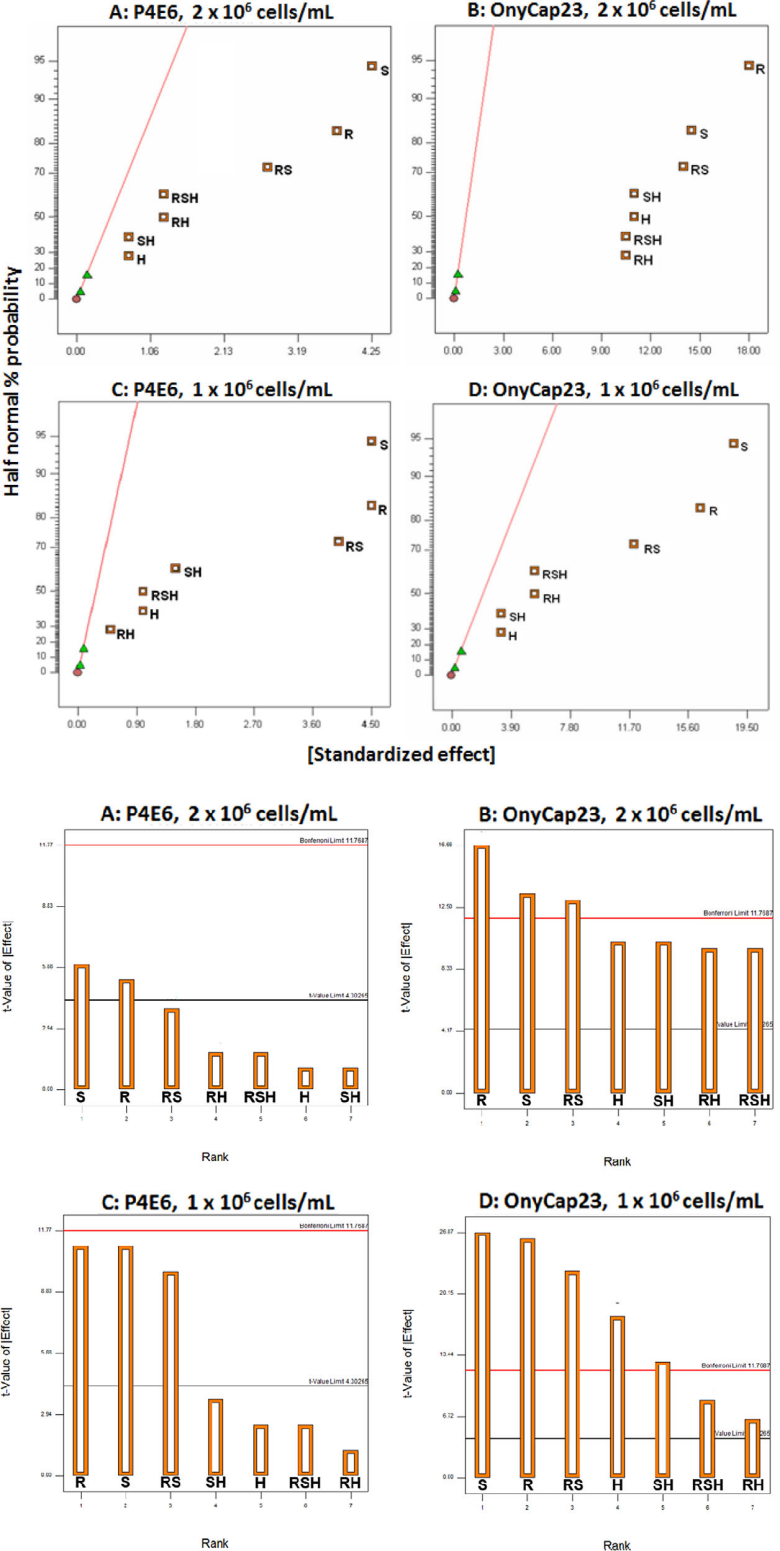
Significance study (top set of figures) and Paretto analyses (lower set of figures) of experimental variables studied on loss of intact cells (see Table I for ranges studied) due to exposure to various combinations of hold time before centrifugation (H), relative centrifugal force (R), and spin time (S). For the significance studies plots, the inclined line is indicative of locus of points where the operating variable or combination of operating variables has no significant effect on cell recovery. The standardized effect is proportional to the square of the effect of operating variable or combination on the resultant loss of intact cells. The corresponding half normal probability values indicate the likelihood that the particular factor or interaction is going to influence that response. For the Paretto plots, the upper bonferroni limit indicates the operating variables or combinations where the statistical confidence of their impact is greater than that for the overall model fit; the lower limit indicates operating variables or combination provide a significant contribution to the overall model fit. The *P* values-of-significance for each DOE-derived effect are as follows:
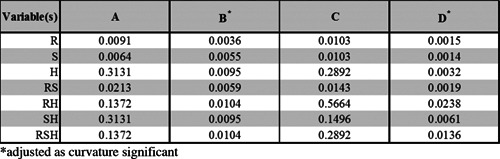
.

**Figure 4:**
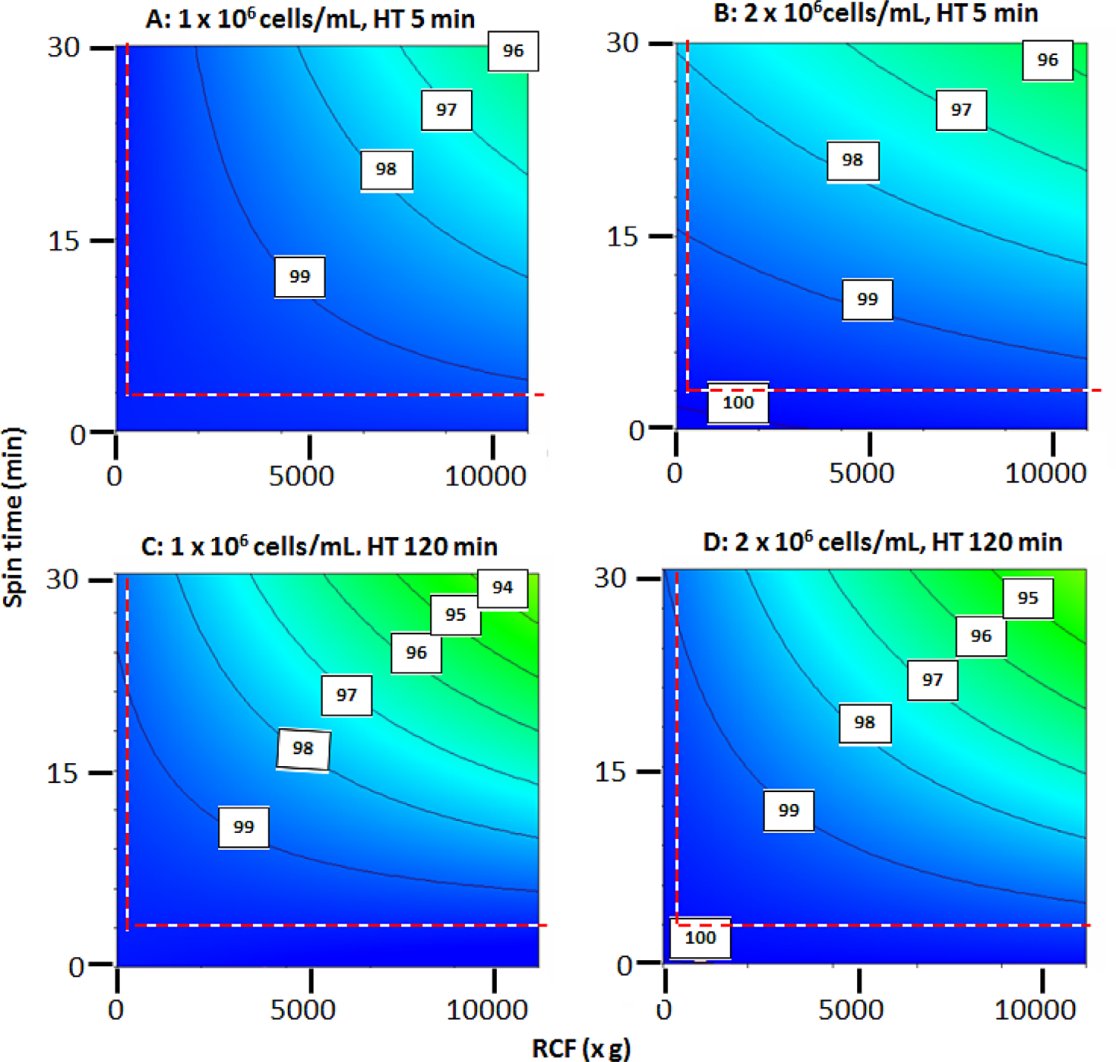
Recovery by centrifugation of P4E6 cells as a function of cell concentration, cell hold time prior to centrifugation, RCF and spin time. DoE relationship values are reported as predicted percentage recovery of intact cells, R_EC_ (equation 1). The resultant model fits incorporating terms relating to all three operating variables and their possible combinations are of high significance level (*P* = 0.02 and *P* = 0.03 for 1 × 10^6^ cells/mL and 2 × 10^6^ cells/mL, respectively. Temperature 21°C, medium CGM. The area to the left and below the red dotted line (R < 250*xg* and S < 3 min) represents data extrapolated from the model created. The effect of increased hold time is an approximately linear proportional decrease in R_EC_ for the equivalent combinations of R and S (relationships not shown here).

**Figure 5:**
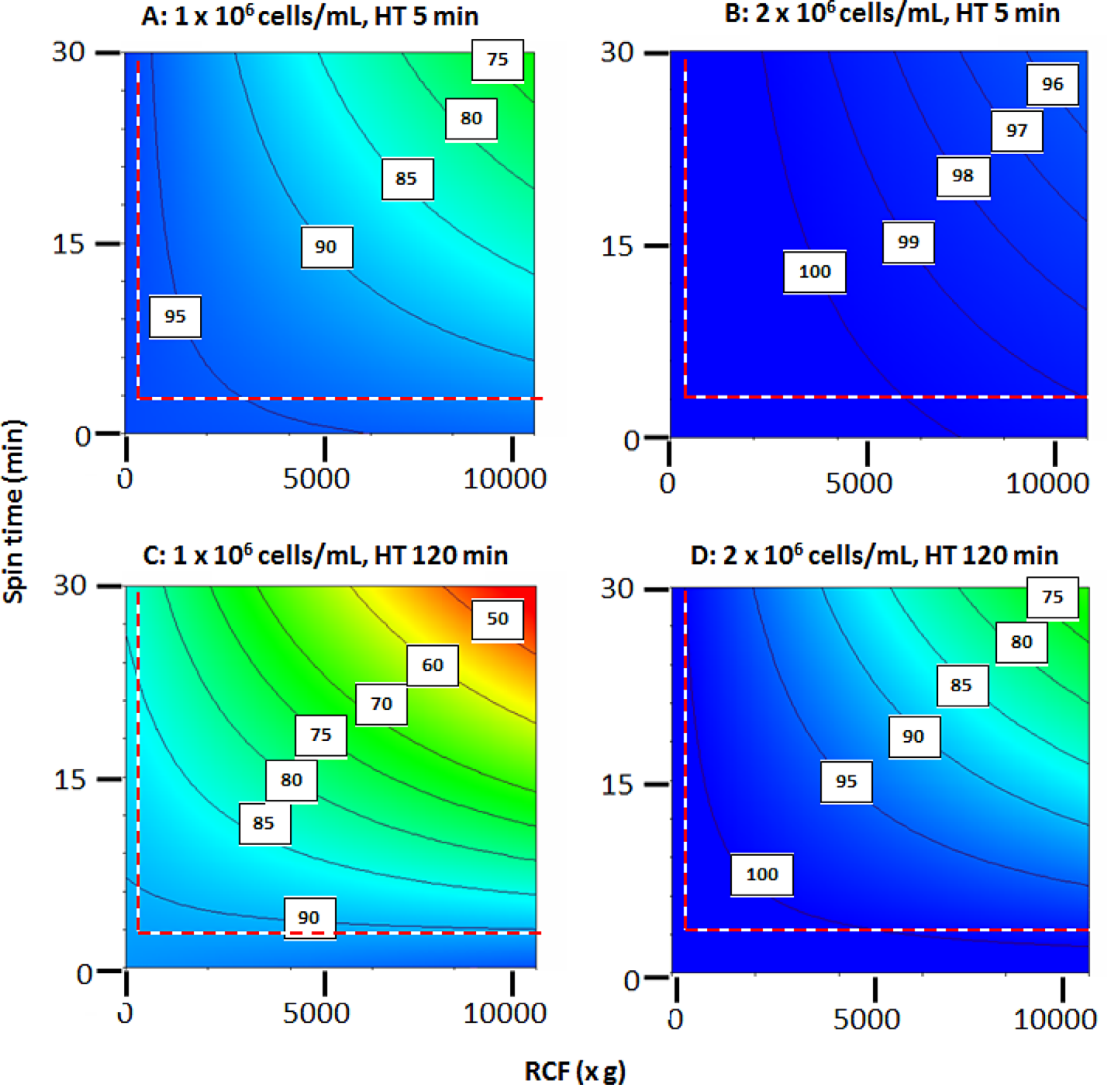
Recovery by centrifugation of OnyCap23 cells as a function of cell concentration, cell hold time prior to centrifugation, RCF and spin time. DoE relationship values are reported as predicted percentage recovery of intact cells, R_EC_ (equation 1). The resultant model fits incorporating terms relating to all three operating variables and their possible combinations are of high significance level (*P* = 0.003 and *P* = 0.02 for 1 × 10^6^ cells/mL and 2 × 10^6^ cells/mL, respectively). Temperature 21°C, medium CGM. The area to the left and below the red dotted line (R < 250*xg* and S < 3 min) represents data extrapolated from the model created. The effect of increased hold time is an approximately linear proportional decrease in R_EC_ for the equivalent combinations of R and S (relationships not shown here).

Figures 3–5 give the visual representation of the output of the DoE studies. Figure 3 presents a significance evaluation alongside a Paretto analysis and *P* values-of-significance for all possible combinations of operating variables. All combinations of the RCF, spin time and hold time before centrifugation are studied to seek evidence for both individual and synergistic effects on cell recovery. For the ranges used, RCF and spin time and the product of these are, in all cases, the strongest determinants of performance. For P4E6 cells at both concentrations the effect of hold time (and combinations involving hold time) are probably statistically insignificant. However, for OnyCap23 cells at both concentrations (Fig. 3B and D) the hold time and various combinations of hold time, RCF, and spin time can also be considered to be significant determinants of performance. It is noted that the behaviour exhibited for high concentration of OnyCap23 (Fig. 3B) is markedly different to that for the other three cases studied; this is attributed to an increased effect of hold time prior to cell concentration and will be discussed later. In order to have a consistent approach to the interpretation of the DoE studies all process variables and their combinations were used in the development of correlations but the emphasis for analysing the results of the correlations derived was based on RCF and spin time and their combination.

The derived contour plots relating the two main variables of RCF and spin time are shown for the two cell lines in Figs. 4 and 5 and the effect of holding time is discussed in the respective legend. The confidence of all the relations derived relating R_EC_ and RCF, spin time and hold time are high (in all cases *P* < 0.03). For P4E6 small but significant increases in level of cell loss occur for greater extents of RCF and spin time used (Fig. 4). The level of cell loss increases slightly when processing cell suspensions of greater age and of lower concentration. In all cases there is an operating window where the combination of RCF and spin time is such that ≥99% recovery of cells is achieved while still achieving modest levels of cell compaction (e.g., RCF of 2500*xg* for 10 min yielding ∼600 × 10^6^ cells/mL—Fig. 2). High levels of compaction of ∼800 × 10^6^ cells/mL are achievable with 4–5% cell loss (RCF 10000*xg* for 30 min) for all start concentrations and hold times used. A similar recovery performance is available for OnyCap23 cells (Fig. 5) but only for fresh cells processed at high concentration (Fig. 4B). For cells previously held for 120 min before processing high recovery levels (R_EC_ > 99%) with modest levels of compaction are still possible but any attempt to achieve high levels of compaction results in very high losses. Operation with either fresh or aged cells at lower cell concentrations results in significant cell loss even under mild centrifugation conditions although it should be noted that 100% cell recovery was recorded at the mildest conditions of 250*xg* for 3 min (Table I).

**Figure 6:**
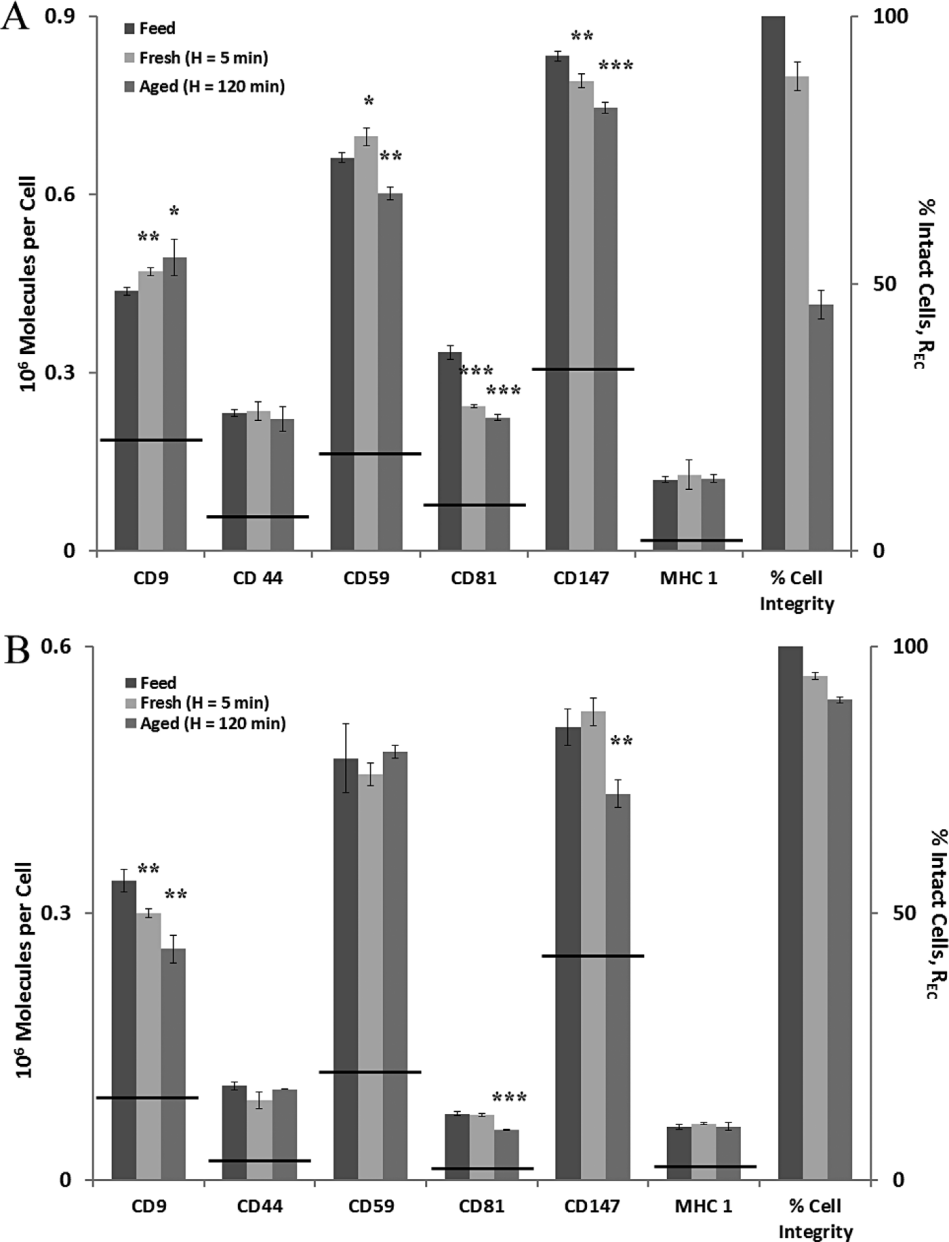
Surface marker analysis. The number of molecules of surface marker per cell for each of the six surface markers investigated for OnyCap23 (A) and P4E6 (B). Both cell lines were processed at 20000*xg* for 30 min after 5 min (▪) and 120 min (▪) hold time, (*n* = 3, +/− 1 sd). Student's *t*-test was conducted on the raw data, comparing processed to control (▪), (^*^P < 0.05, ^**^P < 0.01, and ^***^P < 0.001). Percentage intact cell values are also indicated for the corresponding cell populations. The Onyvax release criteria threshold for each surface marker is represented by the black lines. Cell marker properties: CD9—tetraspanin protein with role in the regulation and modulation of cell development, activation, growth, aggregation, adhesion, and motility (Higginbottom et al., 2003; Ikeyama et al., 1993; Masellis-Smith & Shaw, 1994); CD44—linked to lymphocyte activation, recirculation and homing, hematopoiesis, and tumor metastasis within prostate cell lines (Simon et al., 2009); CD59—protects human blood and vascular cells from injury and lysis (Zhao et al., 1998); CD81—is involved in the immune response with increased expression on T cells during infection; CD147 or Collagenase Stimulatory Factor—co-ordination of cell adhesion with proteolysis, cell communication and signal transduction (Guo et al., 1998; Muramatsu & Miyauchi, 2003; Nabeshima et al., 1991); MHC—major histocompatibility complex molecules involved in the presentation of foreign antigens to the host immune system in order to elicit an immune response (Gruen & Weissman, 1997).

Figure 6 provides one further measure of the impact of centrifugation on critical cell quality attributes, that is, the presence of cell surface molecules. Even at extreme conditions of centrifugation, that is, RCF of 20000*xg*, spin time of 30 min, the high levels of cell recovery observed processing fresh cells is matched by a high level of recovery of a range of surface markers associated with different biological functions (see figure legend for details). Only for the CD59, CD147, and the CD81 markers is there significant and consistent evidence of loss (or down regulation), albeit to a relatively small (∼10%) extent. The levels of loss are much lower than the loss of membrane integrity recorded for OnyCap23. In all other cases, there is no significant difference in marker level except for CD9 where there is both up regulation for OnyCap23 and loss or down regulation for P4E6, but again only to a small (∼10%) extent. As can be seen in Figure 6 the changes in marker level are well below those deemed unacceptable as described in the product release specifications. However, even small changes as recorded here may be an indication of an unacceptable change of the cells if they are to be used for other therapeutic proposes. It should be noted that the marker levels were for cells which had also been freeze—thawed as might occur in a process preparation, for example, for cell therapy preparation in phase between processing and administration or for a vaccine storage (McCoy et al., 2009, 2010) and so may be affected by small extents of lysis occurring during freeze thaw. Surface marker analysis of cells before freezing (e.g., using multiplex analysis to ensure rapid analysis of multiple markers) would be necessary if the effects of centrifugation alone were to be studied but the relatively small changes noted in Figure 6 indicate little change in the centrifugation step alone.

**Figure 7:**
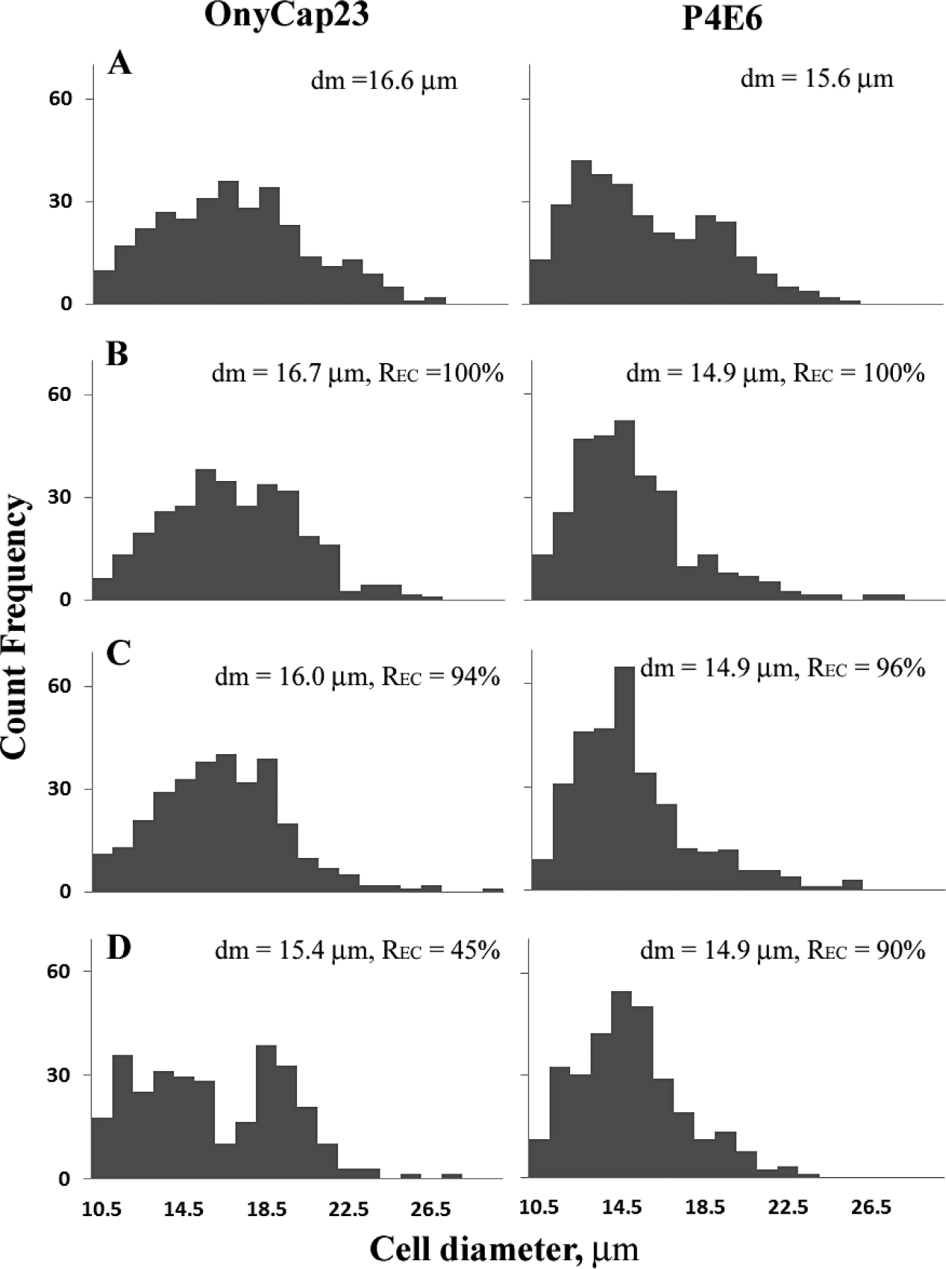
Normalized size distributions (with respect to total cell counts) of OnyCap23 and P4E6; (A) fresh control, (B) hold time (H) = 5 min, centrifugation RCF (R) = 250*xg*, spin time (S) = 3 min, (C) H = 60 min, R = 10000*xg*, S = 30 min, (D) H = 120 min, R = 20000*xg*, S = 30 min. The mean cell diameter, number basis, and the % cell recovery, R_EC_, are given as insets. Temperature = 21°C.

Image analysis of the total cell population of surface attached cells as a result of centrifugation and resuspension show little change in cell morphology (results not shown here). The size distributions of various cell preparations in suspension were analysed by image analysis with the size related to the diameter of a sphere with equivalent projected area. Sample size distributions for P4E6 cells before centrifugation (Fig. 7) show a monomodal distribution with some clumping. Centrifugation and resuspension appears to just disrupt the clumps resulting in similar size distributions for the various conditions studied ranging from gentle centrifugation (RCF = 250*xg* for 3 min) of fresh cells to extreme centrifugation (*RCF* = 20000*xg* for 30 min) of aged cells. Similar results are noted for OnyCap23 cells except for the appearance of a bimodal distribution at extreme centrifugation conditions of aged cells. Of note is the larger size of OnyCap23 cells with an average cell volume 1.2 fold larger than P4E6.

**Figure 8:**
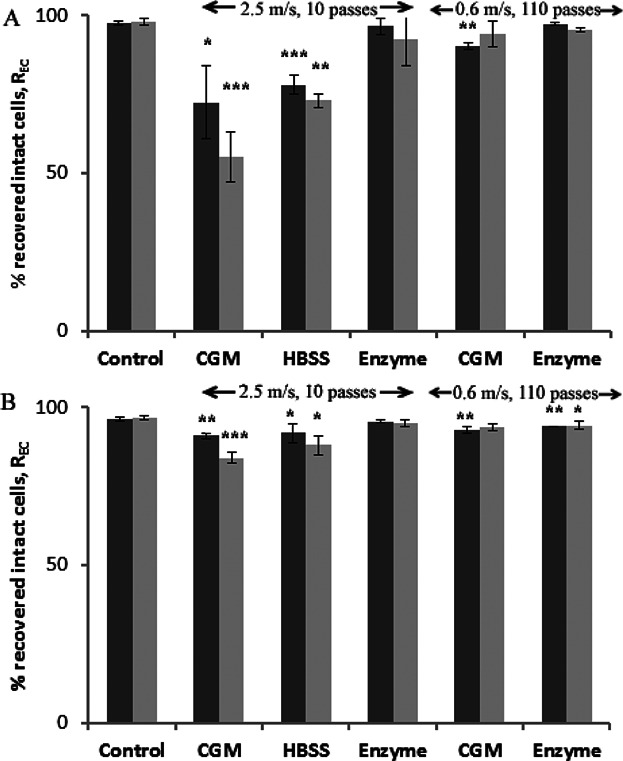
Effect of different resuspension methods. OnyCap23 (A) and P4E6 (B) cell lines were processed at 20000*xg* for 30 min after hold times of (▪) 5 min and (▪) 120 min. For the first two data sets, cells were prepared in CGM (Set 1) or HBSS (Set 2) and resuspended manually at 2.5 m/s. For the remaining data sets cells were respectively prepared in CGM and resuspended automatically at 0.65 m/s, were treated with enzyme and resuspended at 2.5 m/s or at 0.65 m/s. Data presented as mean +/− sd (*n* = 3). Significant loss of recovered intact cells compared with unprocessed cell samples were noted with *P* < 0.05 (*), *P* < 0.01 (**), *P* < 0.001 (***).

One major source of applied shear stress will be during cell pellet resuspension with repeated flow through a pipette tip. To explore the effects of shear stress, the resuspension is studied of sediments prepared using extreme centrifugation conditions (*RCF* = 20000*xg* for 30 min) of fresh (Fig. 8A) and of aged (Fig. 8B) cells. As before, manual resuspension leads to small reduction in recovery of P4E6 cells and a high reduction in recovery of OnyCap23 cells. In both cases cell aging leads to a greater loss. A change in cell environment prior to centrifugation from the protein based cell growth medium (CGM) to a HBSS buffer free of Ca^2+^ and Mg^2+^ results in subsequent higher yields of recovered intact cells. Pre-treatment of the cell pellet to enzymatically digest matrices which will have formed between cells, and hence detach the cells, leads to no cell loss even when using a high flow velocity during cell resuspension. Evidently it is the use of high velocity flow stresses for the initial detachment of the cells within the highly compacted pellet which leads to loss of intact cells. Once the cells are detached and/or freely suspended they appear to be resistant to damage even at the highest flow conditions used during resuspension. The use of lower velocities for cell resuspension, albeit for a much greater number of passes, leads to nearly complete recovery for P4E6 and to small losses (∼4%) for OnyCap23.

**Figure 9:**
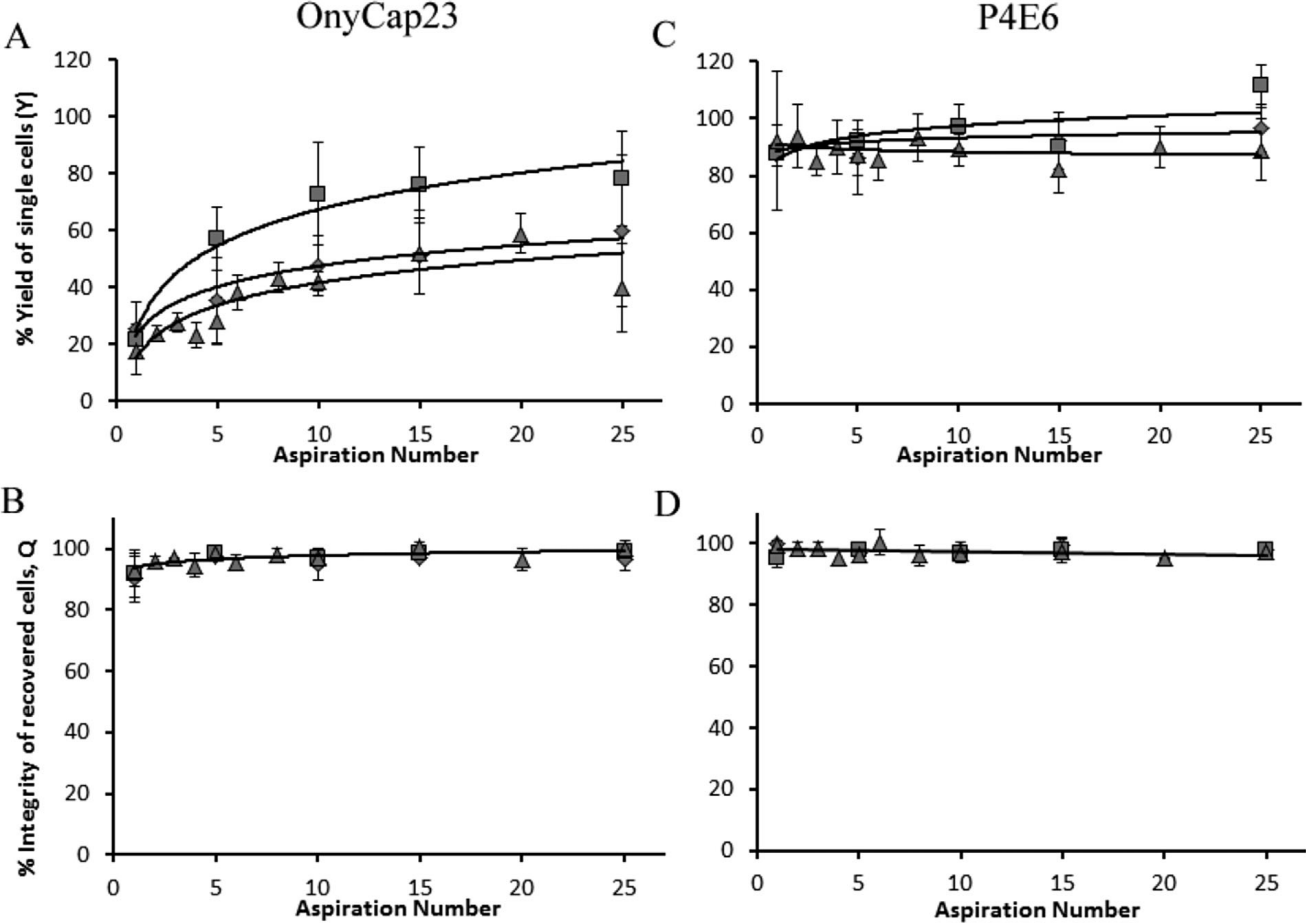
Effect of varying the defined cell resuspension protocol on the recovery of cells from sediment for OnyCap23 (A and B) and P4E6 (C and D). The results are reported as (A and C) single cells in suspension as % total cells in pellet, and (B and D) the % cell integrity of the recovered single cells. Cells were transferred to a 96 well plate and pelleted at either 1500*xg* or 2500*xg* for 5 min. Up to 25 controlled aspiration cycles were then performed using a Tecan automated platform at volumetric flow rates of either 600 μL/s or 900 μL/s. (2500*xg*, 600 μL/s (♦); 2500*xg*, 900 μL/s (▪); 1500*xg*, 600 μL/s (▴). (600 μL/s per transfer of suspension), with the pipette tip located 4.57 mm (i.e., 6× id) above the pellet surface, with subsequent analysis of single cells released into suspension. Each trial conducted in triplicate; data presented as mean +/− sd (*n* = 3).

The ease of cell pellet resuspension into single cell suspensions was studied using an automated platform to allow multiple studies under controlled conditions (Fig. 9). This necessitated using lower RCF values and a narrower pipette diameter than for resuspension studies reported above (Fig. 8). The tip velocities and tip shear rates (∼tip velocity/diameter) used were kept within range of the previous resuspension studies. Cell pellets were subjected to a set number of controlled aspiration/resuspension cycles. P4E6 cells were almost totally resuspended into a single cell suspension after only one controlled resuspension cycle, yielding ∼90% single cells from the total cells originally pelleted. The release of OnyCap23 cells was more gradual. The cells released for both cell lines were of cell integrity consistently above ∼95%. OnyCap23 cell aggregates require considerably more mechanical manipulation to disperse into single cell suspensions than equivalent P4E6 aggregates indicating that cell-cell connections may be stronger within a sedimented OnyCap23 population; perhaps the “stickier” of the two cell lines. Higher rates of resuspension are achieved using greater flow rates. The translation of these observations on cell resuspension to considerations of how large-scale operations might be designed is discussed later.

## Discussion

One key observation in this paper is that it is possible to achieve high levels of cell recovery with membrane structure remaining intact when operating at high relative centrifugal forces (ca 10000*xg*) for long spin times (30 min). This allows high levels of compaction of the cell pellet to be achieved which provides advantages of easier decanting to remove supernatant, reduced carryover of soluble contaminants with the cells and, where the cells are to be used to prepare constructs, cell paste of suitable concentration to mix with scaffold material.

The cell concentrations achieved for P4E6 were typically 1.1 to 1.3 fold higher than for OnyCap23; for example at *RCF* = 10000*xg*, time = 30 min, 600 × 10^6^ cells/mL was obtained for OnyCap23 compared with 800 × 10^6^ cells/mL for P4E6. The 1.2 fold larger cell volume for OnyCap23 compared with P4E6 suggests the same extent of dewatering for both cell suspensions based on residual voidage in the sediment (the residual voidage appears to approach zero but the size distribution data is not specific enough to provide a definite value here; it would be expected that the cells would be flexible enough to pack down and fill nearly all the available space).

A second key observation is that cell damage during centrifugation is determined by the combined effects of the extent of compaction achieved and the method of resuspension used. However, greater compaction in itself does not appear to result in cell damage but rather leads to increased hydrodynamic stress during resuspension. A compacted cell pellet might be expected to have an apparent viscosity, μ, >0.02 N s m^−2^ (Zoro et al., 2009). For a mean flow velocity, v, of 2.5 m s^−1^ in a 1.0 mm id (d) pipette (Fig. 8), a maximum wall stress, τ, of ∼300 N m^−2^ might be estimated (τ = 8 vμ/d). This is of a similar level to the critical stress values of 275 N m^−2^ and 235 N m^−2^ above which damage occurs for P4E6 and OnyCap23 cell lines, respectively (Acosta-Martinez et al., 2010) and possibly explain the cell loss observed (Fig. 8). At 0.65 m/s in a 1.0 mm id tip, *τ* = 70 N m^−2^ which is probably sufficiently lower than the critical shear stress values for the two cell lines to result in the little to no loss of cells observed even for 100+ passes (Fig. 8). The relatively low centrifugation speeds used for cell pellet preparation for the resuspension studies reported in Fig. 9 will yield pellets of lower initial viscosity than those used in Fig. 8 leading to the low levels of cell loss noted despite the high tip velocities and shear rates used.

The greater extent of cell damage observed when processing a feed of lower cell concentration (Figs. 4 and 5) using high degrees of centrifugal compaction suggests that a fixed number of cells might be damaged in a resuspension process, this number becoming increasingly significant as the amount of cells present decreases. The scaling rules determining resuspension of different quantities of cells are yet to be determined. The studies in this paper have focussed on avoiding regimes of possible damage.

It has been suggested elsewhere (Papantoniou et al., 2011) that cell damage during capillary flow induced disruption of embryoid bodies may be due to the sudden detachment of neighbouring cells leading to wall damage in one of the detaching cells. A similar mechanism might also be occurring during the dispersion of the centrifuged cell pellet. The use of enzyme to help disrupt matrices leading to cell-cell interaction was shown to prevent cell damage during release from embryoid bodies during capillary flow. Similarly the use of enzyme digestion prior to resuspension was found to reduce cell loss to negligible levels (Fig. 8) although evidently this leaves the practical dilemma of having to employ a separation technique to remove the enzyme.

One method of reducing cell-cell interaction in the sediment is by changing the cell environment prior to processing. For example surface proteins presented for the formation of cell-cell bonds require the presence of Ca^2+^ to facilitate the mechanisms by which they cluster and present (Umbreit & Roseman, 1975). The replacement of CGM with protein-free Hanks balanced salt solution (HBSS) (without Ca^2+^ and Mg^2+^) led to a reduction in cell loss (Fig. 8) although changes in cell elasticity and porosity as a consequence of an altered osmotic pressure may also alter cell vulnerability to shear forces (Ramirez & Mutharasan, 1992). Keeping hold times of cells to minimum may also reduce the extent of cell-cell interaction (clumping) and hence cell damage (Figs., 5, and 8 4).

Other cell quality indicators studied here included cell surface markers and cell size. For both of these the analysis is of the complete cell suspension, that is, mixes of cells with intact and with disrupted cell membrane. From size distribution studies of cells in suspended form no evidence was found for significant change in size of cells surviving with the membrane intact (Fig. 7). Future studies will examine the potential for cells to continue growing and the resultant morphology. The cell surface marker analysis (Fig. 6) generated similar data as previously reported for shear studies of the same cell lines (McCoy et al., 2009) where loss of cell membrane integrity exceeds considerably any change in the presence of surface markers. Some possible trends are observed for a change in marker content associated with a decrease in cell membrane integrity for: CD9 for both cell lines (but up for OnyCap23 and down for P4E6); CD147 and CD81 (both down); and CD59 (down for OnyCap23 only). Details of the cell markers' function are provided in Figure 6 legend.

The two cell types studied here exhibit different responses to processing stresses and the trends observed here are the same as reported elsewhere for different unit operation stress investigations (Acosta-Martinez et al., 2010). Measurements of the Young's Moduli of different prostate and prostate cancer tissue suggest P4E6 cells have higher surface membrane deformability than cells similar to the OnyCap23 cells; this is as a result of the state of health and degree of metastasis of the P4E6 cells (Faria et al., 2008). This great level of cell elasticity of P4E6 cells along with their smaller size (Fig. 7) are possible contributory factors to the higher levels of recovery of intact cells. It has also been observed that P4E6 cells yield a more tightly packed pellet when centrifuged (Ramirez & Mutharasan, 1990) which might lead to greater damage when considering possible damage due to disruption of cell-cell contacts. However as indicated above at the centrifugation conditions studied the dewatering levels achieved for the two cell lines were broadly similar and the greater size of OnyCap23 cells would lead to greater levels of cell-cell contact to be disrupted on resuspension. This relates to the increased effect of hold time prior to centrifugation especially as observed at higher cell concentration (Figs. 4D and 5D) which might increase the extent of cell-cell interaction. A second interesting observation is the greater susceptibility of the OnyCap23 cell line at a lower start concentration to damage by centrifugation/resuspension. This might be related to protective effects often observed at higher cell concentrations.

### Processing Implications

The performance of a cell recovery process might be determined in terms of the recovery of viable functional cells, the extent of contaminant removal and in some instances the extent of cell concentration achieved. The use of high levels of dewatering to help aid contaminant removal might impact the yield of viable cells unless care is taken over the method of cell resuspension. Low shear stress multipass resuspension has to be achieved without increased chance of contamination in what is an inherently non-enclosed operation. Several alternatives to batch centrifugation exist for cell recovery which may be used in a fully-enclosed mode. These include: (i) the use of porous membranes in cross flow mode under controlled shear to prevent cell damage (Pattasseril et al., 2013; Rowley et al., 2012); (ii) the use of filtration in dead end mode with back flush for recovery (Sowemimo-Coker et al., 2009); (iii) the use of continuous centrifugation (Johnson et al., 1996; Kim et al., 2008); (iv) the use of combined centrifugation and contraflow to band the cells (James, 2011). In all cases the extent of cell concentration achieved is generally low so as to give a cell suspension which flows. However, in situ washing may be used to help achieve contaminant removal. There is evidence for greater cell loss in the enclosed system due to increased potential for hold up of product in the equipment, for example, for cross flow filtration (Rowley et al., 2012). Each of the methods described are still under considerable development with cross-flow membrane techniques probably providing currently the more accessible option for successful cell harvesting if dead-end centrifugation is to be replaced on scale-up, as scale down models exist for development work; for large scale production processes, significant development and application work is occurring within an industrial context with contra-flow centrifugation operating in single-use containers.

The studies within this paper provide a method for determining how dead-end batch centrifugation may be used to yield high levels of dewatering (and hence contaminant removal) and also to retain high levels of intact cell recovery by controlled resuspension. The design of the resuspension process will be highly dependent on the cell type and how the cells interact with each other (Fig. 9). A critical design parameter is the maximum shear stress to which the cells will be exposed and evidently variable speed (for stirred systems) or flow (for plunging jet systems) will allow control of shear stress as the apparent viscosity of the pelleted cells is reduced as the dispersion process proceeds. Application is still likely to require operation in controlled environments, for example, using robotics or careful manual operation but this study does point way for design of integrated centrifugation, decanting and resuspension operations.

An added benefit of the formation of compact cell pastes is the easier operation of the decanting stage without adventitious loss of cells. Also, high dewatering levels leading to fewer wash stages reduces the chance of contamination in this highly manual operation. Finally, for process robustness it also appears that substantial hold-times prior to centrifugation (up to 2 h studied in this paper) are possible without leading to significant cell loss provided the resuspension process is carefully controlled; this provides a valuable contribution to achieving process robustness. Future studies will report on the effect of centrifugation conditions on a range of other aspects of cell quality which might affect their use as a cell therapy. For example for the feed suspensions used in these studies, the proportions of cells which were either early or late apoptotic or necrotic were in each case <5% of the total population (measured by detection of active caspases in combination with membrane permeability using propidium iodide). Studies to be reported (in preparation) demonstrate little to no increase in such populations could be achieved by operation at high RCF (e.g., 10000*xg*) provided care taken over the cell holding time before centrifugation and the method of resuspension with this result being easier to achieve with P4E6 cells compared with OnyCap23 cell lines.

## Conclusions

The use of Design of Experiments methods provides a useful means of exploring the synergistic effects of a range of processing variables to help determine potential windows of operation for robust preparation of cells. The extent of damage of cells during dead-end batch centrifugation is determined primarily by a combination of the extent of exposure to centrifugal forces (product of RCF and spin time) and the method of cell pellet resuspension. This extent of damage is increased by (a) the holding the cells prior to centrifugation (for ∼2 h), (b) operation at lower cell concentration in the feed, and (c) use of increased pipette tip velocities during resuspension. The use of low pipette tip velocities reduces this damage to negligible levels even over the large number (>100) passages needed to effect complete resuspension. The susceptibility to damage (i.e., loss of membrane integrity) is affected by the choice of cell line. Damage as measured by change in presence of cell surface markers is less marked than is observed by loss of membrane integrity for both cell lines studied.
